# Formation Mechanism of Lipid and Flavor of Lard Under the Intervention of Heating Temperature via UPLC-TOF-MS/MS with OPLS-DA and HS-GC-IMS Analysis

**DOI:** 10.3390/foods14142441

**Published:** 2025-07-11

**Authors:** Erlin Zhai, Jing Zhang, Jiancai Zhu, Rujuan Zhou, Yunwei Niu, Zuobing Xiao

**Affiliations:** 1 School of Perfume and Aroma Technology, Shanghai Institute of Technology, No. 100 Haiquan Road, Shanghai 201418, China; zhaierling20@163.com (E.Z.); zhangj@sit.edu.cn (J.Z.); zjc@sit.edu.cn (J.Z.); zhourujuan@sit.edu.cn (R.Z.); 2 School of Agriculture and Biology, Shanghai Jiao Tong University, Shanghai 200240, China

**Keywords:** lard, lipid, volatile compounds

## Abstract

Lard imparts unique organoleptic properties that underpin its essential role in Chinese gastronomy; however, the specific lipid precursors contributing to its aroma remain unclear. This study explores the flavor formation mechanism of lard by comparing its texture and aroma at two preparation temperatures, 130 °C and 100 °C. We identified a total of 256 and 253 lipids at these temperatures, respectively, with triacylglycerols (TGs) and diacylglycerols (DGs) being the predominant lipid species. An HS-GC-IMS analysis detected 67 volatile compounds, predominantly aldehydes, acids, and alcohols. A subsequent Orthogonal Partial Least Squares-Discriminant Analysis (OPLS-DA) identified 49 discriminatory lipids and 20 differential volatiles. A correlation analysis showed a positive relationship between aldehydes and unsaturated triglycerides in lard, with TG (16:1-16:1-18:0), TG (17:2-18:1-18:1), TG (16:1-17:1-18:1), and TG (18:1-18:1-20:1) identified as characteristic markers at both temperatures. Furthermore, there was a positive correlation between ketones and alcohols and phospholipids and sphingolipids containing unsaturated fatty acid chains. TGs and glycerophospholipids (GPs), rich in polyunsaturated fatty acids, are likely key precursors driving the formation of distinct flavors during lard processing. This study elucidates the mechanistic interactions between lipids and volatile organic compounds, providing a framework for optimizing lard processing protocols and flavor modulation.

## 1. Introduction

Lard, a conventional cooking fat, contains abundant essential fatty acids, including arachidonic acid, as well as various fat-soluble vitamins vital for human health. It has a low content of trans fatty acids and a relatively high melting point (28–48 °C) [1]. Its unique flavor and wide plasticity range [2] make it a preferred ingredient in the food industry, especially in baked goods, such as crackers and bread, as well as in shortening production [3,4,5].

Lard’s physicochemical characteristics and compositional integrity are critically determined by the extraction methodology employed. Traditional extraction techniques include dry and wet rendering. Dry rendering, carried out at 115–140 °C without water, facilitates easier separation and extrusion of lard residues, producing a darker product with more intense flavor compounds [6]. In contrast, wet rendering involves processing with water at around 100 °C, resulting in a milder, more neutral flavor due to gentler conditions. Although newer methods such as enzymatic and ultrasound-assisted extraction have been developed [7], dry and wet rendering remain the dominant techniques in lard production. These methods yield lard with distinct flavor profiles and physicochemical characteristics, which influence consumer preferences and market competitiveness. Dry-rendered lard, known for its strong flavor, is widely used in Chinese cuisine, while wet-rendered lard—with its subtler taste—is better suited for products requiring less pronounced flavor, such as shortenings and pastries. Wang et al. reported that dry-rendered lard had a higher acid value than wet-rendered lard, although differences in raw materials and processing methods were also noted [8].

Food flavor profiling routinely employs hyphenated techniques, including electronic nose, GC-MS, and GC-O-MS technology for compound identification and sensory correlation [9]. Lipid composition governs aromatic compound formation, with specific fatty acid profiles dictating volatile generation pathways. Gas chromatography–ion mobility spectrometry (GC-IMS) has recently become popular for analyzing and monitoring volatile flavor compounds in food thanks to its advantages: no sample pretreatment required, high sensitivity, rapid detection, excellent separation efficiency, and visualized results [10,11,12]. Thermal processing of lard induces hydrogen abstraction from unsaturated fatty acid alkyl chains, triggering oxidation pathways that yield hydroperoxides as primary reaction products [13]. These hydroperoxides undergo spontaneous decomposition, generating organic compounds such as ketones, aldehydes, carboxylic acids, alcohols, esters, and hydrocarbons [14]. Xu et al. [15] identified unsaturated aldehydes as the main volatile compounds produced during lard oxidation. Key flavor components in heat-treated lard include hexanal, (E)-2-heptenal, pentanal, and nonanal [16,17,18].

Lard is primarily composed of glycerides, and its lipid precursors can generate volatile flavor compounds through Maillard reactions and lipid oxidation. Fatty acid saturation indices and concentration profiles govern volatile compound formation [19]. Lipidomic has been widely applied in food science for the identification and comprehensive analysis of lipids [20,21], enabling deeper insights into the roles and potential mechanisms of lipid molecules in biological processes [22]. Previous studies have identified triglycerides such as TG (16:0-18:1-18:1), TG (16:0-16:1-18:1), and TG (16:0-18:1-18:2) as important precursors in flavor formation in beef [23], and potentially in the aroma of roasted pork as well [24]. However, comparative studies on lipid composition between dry- and wet-rendered lard are limited, and research on how lipid composition changes relate to flavor compound formation remains scarce. Much of the existing work has focused on the identification of final volatile flavor substances or the overall nature of lard from a single extraction method. There is a lack of in-depth, quantitative correlation studies on the lipid molecules themselves that dominate the composition of lard, especially the systematic differences in the specific TG species and compositions in dry- and wet-rendered lard, and how they serve as the core precursors that drive the subsequent oxidation process and ultimately determine the formation of the characteristic flavor profile.

In this research, lipid composition and volatile substances in lard were characterized using ultra-performance liquid chromatography combined with quadrupole time-of-flight mass spectrometry (UPLC-Q-TOF-MS) and headspace gas chromatography–ion mobility spectrometry (HS-GC-IMS). By multivariate statistical analysis, we systematically investigated the differences in volatile components and identified key differential compounds. We will seek to establish a robust scientific foundation and a novel analytical approach for enhancing the precision of lard manufacturing, objectively assessing product quality, and creating tailored products to meet specific flavor criteria.

## 2. Materials and Methods

### 2.1. Materials and Reagents

Abdominal adipose tissue from Great Yorkshire pigs, sourced from Henan Province (Zhengzhou, China) was collected and stored at −20 °C. 1,2-Dichlorobenzene (99%, used as an internal standard) and a series of n-alkanes (C6–C25, for retention index calibration) were purchased from Sigma-Aldrich (St. Louis, MO, USA). Ether, potassium hydroxide, isopropanol, potassium iodide, chloroform, acetic acid, phenolphthalein, and sodium thiosulfate were obtained from Shanghai Aladdin Biochemical Technology Co., Ltd. (Shanghai, China).

### 2.2. Preparation of Lards

Different lard rendering technology:

For the dry rendering method, adipose tissue was minced into 1 cm^3^ pieces and simmered (130 ± 5 °C, 20 min) on an induction heating platform with continual manual agitation. The rendered fat was then cooled to 55 °C, and the liquid portion was collected and stored at −20 °C.

For the wet rendering method, adipose tissue was sectioned into 2-cm^3^ cubes, thermally processed in water (1:1 mass ratio) at 100 °C under atmospheric pressure, with subsequent fat isolation via solid-residue filtration through medical gauze. Each method was performed in triplicate to ensure reproducibility.

### 2.3. Determination of Peroxide Value (PV), Acid Value (AV), and Water Content

Based on safety control considerations during lard rendering, the PV, AV, and water content of the lards were conducted according to the method in [25].

### 2.4. HS-GC-IMS Analysis of Lard

Accurately transfer 2.0 ± 0.1 g of the sample into a 20 mL headspace vial. Add 10 μL of a 100 ppm 2-methyl-3-heptanone internal standard solution as a spike. Prior to performing triplicate GC injections, equilibrate the vial at 60 °C for 20 min to ensure uniform distribution of volatile components.

The sample was then put through an incubation process at a temperature of 60 °C for 20 min. The system received an injection of 500 μL during this step, and the injection was performed in non-split mode. During incubation, the shaking speed was kept at 500 rpm and the injection needle was maintained at a constant temperature of 85 °C. The column was maintained at a constant temperature of 60 °C during the chromatographic analysis, and high-purity nitrogen gas (≥99.999%) was used as the carrier gas. The flow rate programmer was as follows: initially held at 2.0 mL/min for 2 min; increased linearly to 10.0 mL/min over 8 min; increased further linearly to 100.0 mL/min over the next 10 min; and finally held at 100.0 mL/min for the final 10 min. The total runtime of the chromatographic analysis was 30 min, with an inlet temperature of 80 °C.

Ionization was achieved using a tritium (^3^H) source. The migration tube measured 53 mm in length and operated at an electric field strength of 500 V/cm. The tube was kept at a temperature of 45 °C and a flow rate of 75.0 mL/min of high-purity nitrogen (≥99.999%) was used as the drift gas in positive ion mode. Retention indices were calculated based on retention time and identified using the GC retention index database (NIST 2014) integrated into Vocal software (2024, 0.4.10), in combination with IMS migration time for compound comparison. Target compounds were analyzed using the Gallery Plot, and Dynamic PCA plug-ins within the Vocal data processing suite. Each sample was performed in triplicate to ensure reproducibility.

### 2.5. Sensory Evaluation Method of Lard

A sensory evaluation panel comprising five female and five male members from the Shanghai Institute of Technology assessed the aroma characteristics of lard prepared using different rendering methods. Aroma attributes—including deep-fat frying, fatty, roasted, earthy, and fecal notes—were described and scored according to the methodology outlined in [17]. All evaluations were conducted in a standardized sensory evaluation laboratory.

Following randomization via 3-digit codes, lard samples in amber-sealed vials underwent 30 min headspace equilibration at 50 °C. A quantitative descriptive analysis (QDA) was performed as follows: panelists scored aroma attributes (10-point intensity scale) through sequential monadic presentation. Inter-replicate validation was conducted.

### 2.6. Electronic Nose Evaluation Method for Lard

Weigh out precisely 5 g of lard into a 20 mL headspace vial, following placement on the e-nose sampling tray, and commence the analysis under the following controlled operational conditions: headspace incubation 60° for 20 min, oscillation speed of 500 r/min, total signal acquisition time of 110 s, and signal frequency of 0.01 s. Dry air was used as the carrier gas, with an injection volume of 5.0 mL at a rate of 125 µL/s and a flow rate of 1 µL/min. The response values from 18 sensors, which reflect the aromatic characteristics of the sample, are detailed in Table S1.

### 2.7. Determination of Lipid Composition for Lard

Vortex 20 mg lard with 200 μL H_2_O and 480 μL MTBE/MeOH (5:1, *v*/*v*) for 2 min in a glass tube. Vortex for 30 s, sonicate in 12–14 °C bath for 10 min, then incubate at –40 °C for 1 h. Centrifuge at 3000 rpm for 15 min at 4 °C. Vacuum-dry 300 μL of the supernatant at a low temperature, then reconstitute the dried sample in 4 mL of resuspension buffer (DCM: MeOH: H_2_O = 60:30:4.5, *v*/*v*/*v*). Vortex the sample in ice water for 30 s and sonicate for 10 min. The centrifuge should be run again at a temperature of 4 °C and a rotational speed of 12,000 rpm, then transfer the supernatant to a new glass vial for LC/MS analysis. Prepare a quality control sample by combining aliquots of the supernatant from each sample and mixing thoroughly.

Lipid profiling was conducted using an Orbitrap Explores 120 mass spectrometer (Thermon Fisher Scientific) coupled to a Vanquish ultra-high-performance liquid chromatography (UHPLC) system from the same manufacturer. Chromatographic separation was achieved on a Phenomenex Kinetics C18 column (2.1 × 100 mm, 2.6 μm particle size). The mobile phase consisted of solvent A: 10 mM ammonium format in a water/acetonitrile mixture (6:4, *v*/*v*), and solvent B: 10 mM ammonium format in isopropanol/acetonitrile (9:1, *v*/*v*). Electrospray ionization (ESI) settings included a sheath gas flow rate of 30 arbitrary units, auxiliary gas at 10 units, a capillary temperature of 320 °C, and a spray voltage of ±3.8 kV in positive mode and –3.4 kV in negative mode. The full scan MS was operated at a resolution of 60,000, whereas the MS/MS resolution was set at 15,000. Stepped normalized collision energy (NCE) values of 15%, 30%, and 45% were applied during the analysis.

### 2.8. Statistical Analysis

The mean and standard deviation of the results from the two samples tested three times were calculated. Data were analyzed using SPSS 2022 (*p* < 0.05). Origin 2022 and MetaboAnalyst 6.0 facilitated multivariate analyses, specifically OPLS-DA modeling and (Variable Importance in Projection) VIP metric calculation. Pearson correlation coefficients quantified lipid–aroma compound relationships. Hiplot was used to generate heat maps and related maps (https://hiplot.com.cn/).

## 3. Results and Discussion

### 3.1. Critical Quality Parameters of Lard: Acid Value, Peroxide Value, and Water Content

The preparation of lard is highly dependent on processing temperature. According to oil and fat standards [26], to ensure oil quality, specific limits are set for AV, PV, and moisture content: AV must be ≤2.5 mg/g, PV ≤ 0.2 g/100 g, and moisture ≤ 0.2%. As shown in Table 1, the lard samples analyzed meet these requirements. The AV reflects the free fatty acid content, which mainly results from the hydrolytic or oxidative breakdown of triglycerides in fats and oils. The PV measures the primary oxidation products and serves as a key indicator of the initial oxidation state; a higher PV indicates more extensive oxidation [27]. Significant differences (*p* < 0.05) in both AV and PV were observed in DL (processed at 130 °C) compared to WL (processed at 110 °C) This is attributed to the increased reactivity of polyunsaturated fatty acids at elevated temperatures, where free radicals induce hydroperoxide formation, thus raising the peroxide value [28]. Additionally, higher temperatures accelerate triglyceride hydrolysis, leading to increased free fatty acids and, consequently, a higher AV [29]. These findings are consistent with Guo’s study on tallow extraction [30] and Liu’s research on pomegranate seed oil extraction [31].

### 3.2. HS-GC-IMS Analysis of Lard

#### 3.2.1. HS-GC-IMS Spectrum Analysis

**Figure 1:**
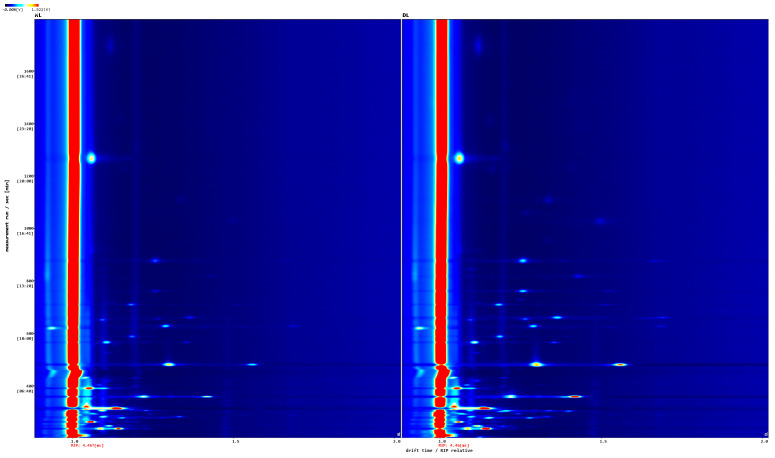
HS-GC-IMS top view of sample spectrum.

To compare the volatile components of lard processed under different conditions, a two-dimensional topographic plot with a blue background was generated using HS-GC-IMS (Figure 1). A red vertical line at 1.0 on the horizontal axis indicates the reactive ion peak (RIP, normalized). Each spot near the RIP corresponds to a volatile compound, with the presence, absence, and color intensity of these spots reflecting the degree of accumulation or degradation. The color gradient from white to red corresponds to an increasing concentration. The plot shows that most volatile compounds have retention times between 200 and 900 s and drift times ranging from 1.0 to 1.6. Certain volatile compounds are shared between lard processed at both 130 °C and 110 °C. Differences in signal intensities at these temperatures primarily arise from thermal degradation and oxidation reactions occurring during processing.

#### 3.2.2. HS-GC-IMS Fingerprint Spectrum

**Figure 2:**
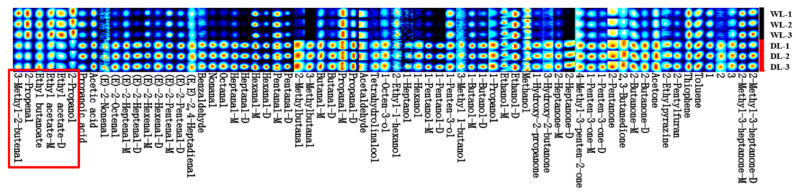
Volatile organic compounds (VOCs) fingerprint of lard. Compounds labeled with “M” and “D” represent the monomeric and dimeric forms, respectively.

The visual fingerprint of volatile compounds provides an intuitive and quantitative comparison of differences between samples. The results are shown in Figure 2, where each row represents a sample and each column corresponds to a volatile component signal peak. “M” and “D” indicate the monomer and dimer forms of the same compound, respectively, while numbers refer to unidentified substances. The brightness and size of each color block are proportional to the peak intensity of the volatile compound, with colors ranging from dark to light to reflect decreasing intensity. Figure 2 reveals that a total of 67 compounds were identified across the different processing methods, including 64 known and 3 unknown compounds. Among these, 34 compounds were solely observed as monomers, while 15 existed in both monomeric and dimeric forms. Despite similar overall profiles, the volatile compound levels varied between the two samples. Specifically, the wet-rendered lard (WL), prepared at 100 °C, showed higher signal intensities for six substances compared to the dry-rendered lard (DL) prepared at 130 °C within a red rectangle in Figure 2. Conversely, DL had higher levels of 54 compounds, including 2 heterocyclic compounds, 25 aldehydes,11 alcohols, 12 ketones, and 4 others. Notably, total signals for aldehydes and alcohols—key contributors to flavor and aroma—were more pronounced in lard prepared at 130 °C than at 100 °C.

#### 3.2.3. Qualitative and Quantitative Analysis of HS-GC-IMS

Volatile flavor compounds in lard heated at 130 °C and 110 °C were analyzed using HS-GC-IMS, focusing on GC retention times and ion migration times. As shown in Table 2, 67 volatile organic compounds were detected within the C2-C10 range, including 27 aldehydes, 16 alcohols, 12 ketones, 3 esters, 2 acids, 1 pyrazine, 1 furan, and 5 other compounds (3 of the 5 compounds have not been identified). Aldehydes, alcohols, and acids were the most abundant, accounting for 50.48%, 24.38%, and 12.39% of the DL at 130 °C, and 42.01%, 29.32%, and 14.2% of the WL at 110 °C, respectively. IMS discriminates electronegative analytes and Brønsted bases, with particular responsiveness to conjugated carbonyl volatile organic compounds [32], allowing differentiation between compound forms like monomers and dimers. Significant differences were observed in most volatile components, except for seven compounds—propionic acid, acetic acid, 2-ethyl-1-hexanol, ethanol-D, 3-methyl-1-butanol, methanol, and thiophene—which showed no significant variation (*p* > 0.05) between the two processing methods.

Aldehydes, which result from the auto-oxidation of unsaturated fatty acids, were found in higher concentrations in lard heated to 130 °C (1473.25 ± 11.8 μg/100 g) than at 110 °C (925.33 ± 9.09 μg/100 g) (*p* < 0.001). The primary unsaturated fatty acids in lard are oleic, linoleic, and linolenic acids [33]. During oleic acid oxidation, four hydroperoxides (8, 9, 10, and 11) form, with hydroperoxides 10 and 11 breaking down into nonanal and octanal, respectively [34]. The nonanal content in DL at 130 °C (25.4 ± 0.58 μg/100 g) was significantly higher than at 110 °C (9.77 ± 0.71 μg/100 g) (*p* < 0.001). Similarly, octanal content at 130 °C (17.39 ± 0.33 μg/100 g) exceeded that at 110 °C (4.43 ± 0.35 μg/100 g) (*p* < 0.001). Linoleic acid undergoes auto-oxidation to form 11- and 13-linoleic acid hydroperoxides. The 11-hydroperoxides decompose into heptanal and (E)-2-octenal, while the 13-hydroperoxides cleave into pentanal and (E)-2-heptenal [35]. The oxidation of linolenic acid produces 12-, 13-, and 16-linolenic acid hydroperoxides. (E, E)-2,4-Heptadienal, a cleavage product of 12-linolenic acid hydroperoxides and a major contributor to lard aroma, was significantly higher in DL at 130 °C (13.04 ± 0.27 μg/100 g) than at 110 °C (11.07 ± 0.46 μg/100 g) (*p* < 0.01). (E)-2-Hexenal derives from cleavage of 13-linolenic acid peroxide, while propionaldehyde originates from 16-linolenic acid peroxide. These unsaturated aldehydes further oxidize to form short-chain aldehydes, such as 3-methylbutanal [36]. DL contained significantly more octanal (16.84 ± 0.66 μg/100 g) than WL at 110 °C (0.69 ± 0.04 μg/100 g) (*p* < 0.001). Given their low aroma thresholds and high volatility, aldehydes contribute notably to oil flavor even at low concentrations. C3-C4 aldehydes typically emit a strong pungent odor, while C5-C9 aldehydes provide clean, oily, and fatty aromas, consistent with findings by Song [37] on lard and Yao et al.’s review of meat aroma compounds. Variations in these components likely contribute to the flavor differences between the two types of lard [38].

Alcohols, important volatile flavor compounds, are generated through the oxidation of fatty acids and the decomposition of secondary hydroperoxides [39]. The total alcohol content in DL at 130 °C (711.57 ± 7.95 μg/100 g) was significantly higher than in WL at 100 °C (645.86 ± 3.73 μg/100 g) (*p* < 0.01). Among the identified alcohols, no significant differences (*p* > 0.05) were observed for 2-ethyl-1-hexanol, ethanol-D, ethanol-M, 3-methyl-1-butanol, and methanol. However, highly significant differences (*p* < 0.01) were found for 1-octen-3-ol, 1-heptanol, 1-hexanol, and 1-butanol-D. Even more pronounced differences (*p* < 0.001) were observed for tetrahydrolinalool, 1-pentanol-M, 1-pentanol-D, 1-pentan-3-ol, 1-butanol-M, 1-propanol, and 2-propanol. The formation pathways include 10-oleate hydroperoxides producing 1-heptanol and 1-octanol, while linoleic acid and palmitoleic acid hydroperoxides lead to 1-pentanol formation. Notably, 1-octen-3-ol, which is derived from linoleic acid oxidation, has a low odor threshold of 1 μg/kg and significantly influences food flavor. Its concentration in DL at 130 °C (8.58 ± 0.56 μg/100 g) was markedly higher than in WL at 100 °C (4.36 ± 0.69 μg/100 g) (*p* < 0.01). This compound is commonly detected in oil-rich foods, imparting mushroom, rose, and hay-like aromas. Meanwhile, 1-hexanol contributes musty, sweet, and woody notes, and 1-pentanol provides fuel-oil-like, sweet, and balm aromas [40].

Furan compounds form during oil oxidation. 2-Pentylfuran, produced via the cyclization of 9-hydroperoxide with an alkoxy group, has an odor threshold of 6 μg/kg and contributes earthy, green, and vegetable-like aromas [41]. In DL prepared at 130 °C, 2-pentylfuran content (5.06 ± 0.16 μg/100 g) and 2-ethylpyrazine content (3.22 ± 0.02 μg/100 g) were significantly higher than in WL at 100 °C (2.5 ± 0.21 μg/100 g and 2.71 ± 0.12 μg/100 g, respectively). The difference in 2-pentylfuran was extremely significant (*p* < 0.001), while 2-ethylpyrazine showed a highly significant difference (*p* < 0.01). 2-Ethylpyrazine emits nutty, woody, potato-like, roasted, and meaty aromas, whereas 2-pentylfuran has bean-like, fruity, soil, green, and vegetable notes. These sensory properties are consistent with electronic nose results.

Ketones mainly arise from esterification reactions between alcohols and free fatty acids during fat oxidation. Most ketones impart floral and fruity aromas and have relatively low odor threshold concentrations. The ketone content in DL at 130 °C was 150.56 ± 1.41 μg/100 g, significantly higher than 97.37 ± 0.67 μg/100 g in WL at 100 °C. Key ketones such as 2-pentanone, 2-heptanone, 2,3-butanedione, and 2-butanone contribute importantly to the characteristic flavors of animal and vegetable oils [42].

Ethyl butyrate and ethyl acetate, the two esters detected, are primarily formed by the esterification of ethanol with acetic and butyric acids, providing fruity and sweet aromas [43]. The content of ethyl butyrate (5.87 ± 0.18 μg/100 g) and ethyl acetate (monomer: 29.55 ± 1.55 μg/100 g, dimer: 10.16 ± 0.86 μg/100 g) in WL at 100 °C was significantly higher than that in DL at 130 °C (5.27 ± 0.22 μg/100 g and 24.41 ± 0.66 μg/100 g, 7.45 ± 0.26 μg/100 g, respectively).

#### 3.2.4. PCA and OPLS-DA Analysis of HS-GC-IMS

**Figure 3:**
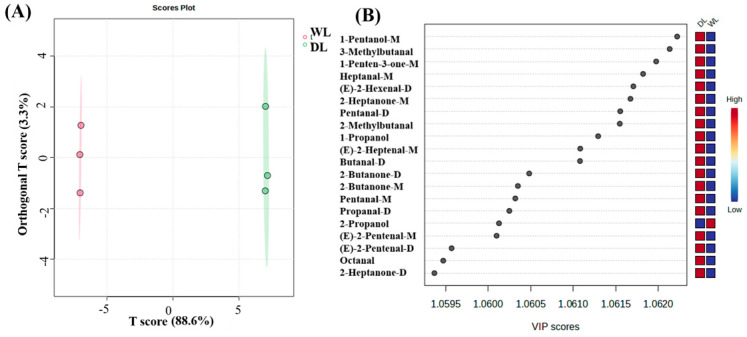
Identification and analysis of volatiles in DL and WL by HS-GC-IMS. (**A**) OPLS-DA plot [1]; (**B**) VIP scores.

Fingerprinting alone inadequately differentiates the volatile components in lard processed by various methods, making it difficult to precisely identify those responsible for sample variation. To effectively discern flavor differences between lard processed at 130 °C and 110 °C, multivariate statistical analyses were applied. The OPLS-DA model established correlations between compound expression and sample groups. As shown in Figure 3A, the PC1 and PC2 accounted for 88.6% and 3.3% of the variance, respectively. This resulted in distinct clustering of samples prepared at 130 °C and 110 °C, indicating significant compositional differences. The VIP analysis (Figure 3B) identified 20 metabolites with significant differences between the groups, including 1-pentanol-M, 1-penten-3-one-M, 3-methylbutanal, heptanal-M, 2-heptanone-M, (E)-2-hexenal-D, 2-methylbutanal, pentanal-D, 1-propanol, (E)-2-heptenal-M, 2-butanone-D, butanal-D, 2-butanone-M, pentanal-M, propanal-D, 2-propanol, (E)-2-pentenal-D, (E)-2-pentenal-M, octanal, and 2-heptanone-D.

### 3.3. Sensory Analysis of Lard

**Figure 4:**
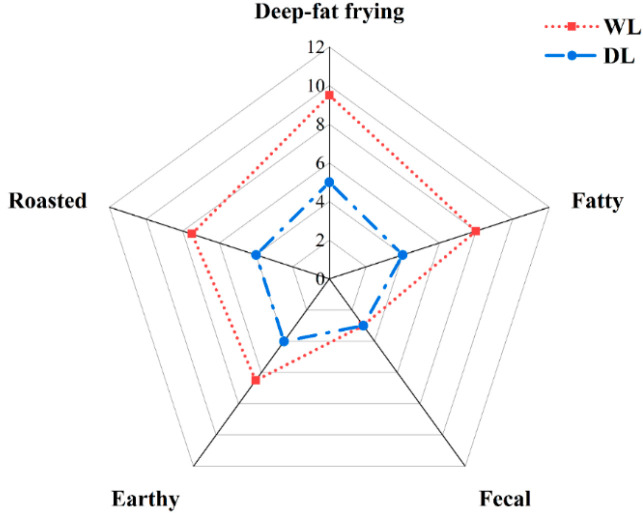
Sensory evaluation analysis of DL and WL.

Flavor plays a critical role in shaping consumer preferences and determining product applications [44]. The lard temperature significantly modifies its aroma profile. As shown in Figure 4, lard processed at 130 °C demonstrated significantly stronger overall aroma, frying, and baking notes compared to WL processed at 100 °C. This difference originates from triglyceride hydrolysis and oxidation during extraction, which release free fatty acids. Unsaturated fatty acids undergo oxidation to form primary and secondary peroxides, which subsequently decompose into aldehydes, ketones, alcohols, and other flavor-active compounds. Elevated temperatures accelerate the generation of these volatile compounds [45].

Among the key flavor compounds identified in lard are aldehydes, including nonanal, hexanal, (E)-2-octenal, and (E)-2-heptenal, which contribute green and fatty aromas; alcohols like 1-octen-3-ol, noted for mushroom and greasy scents; γ-lactone, imparting a deep-frying aroma; and 2-pentylfuran, associated with baking notes. The concentration of these compounds increases with processing temperature, consistent with the HS-GC-IMS analysis. Both lard samples processed at 130 °C and 110 °C showed only mild “odor” intensity, with no significant difference between them. Unique to lard are volatile compounds such as 3-methylindole and indole, which produce undesirable fecal odors. These arise from microbial decomposition of tryptophan in pig intestines and accumulate in adipose tissue via circulation. These lipophilic compounds were not uncovered by HS-GC-IMS, likely due to methodological differences in detection.

### 3.4. Analysis of Electronic Nose Results

**Figure 5:**
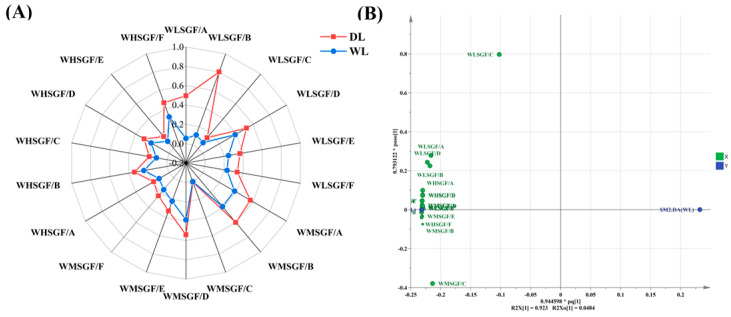
Electronic nose discrimination of DL and WL. (**A**) Radar chart of aroma attributes. (**B**) Loading plots for OPLS-DA analysis.

The electronic nose is a rapid and objective analytical tool for profiling volatile odor substances through sensor arrays combined with pattern recognition technology, playing a vital role in aroma analysis [46]. It has been successfully applied to distinguish odor profiles in tea [47], meat [48], fish [49], and various other substances. An electronic nose was utilized to characterize the volatile signatures of lard samples processed under different thermal conditions. The flavor radar diagram is shown in Figure 5A. It reveals that the response values of the DL (lard processed at 130 °C) to 18 sensors (the response values from 18 sensors are detailed in Table S1) exhibited higher response values than those of the WL (lard processed at 100 °C). Except for the WLSGF/C sensor, which showed no significant difference (*p* > 0.05), all other sensors exhibited varying degrees of significance. Specifically, WLSGF/A showed significant differences (*p* < 0.05); WLSGF/B, WLSGF/D, WMSGF/B, WMSGF/C, WMSGF/E, WMSGF/F, WHSGF/C, and WHSGF/E showed highly significant differences (*p* < 0.01); and WLSGF/E, WLSGF/F, WMSGF/A, WMSGF/D, WHSGF/A, WHSGF/B, WHSGF/D, and WHSGF/F showed extremely significant differences (*p* < 0.001). These results indicate that heating lard at a higher temperature of 130 °C generates higher concentrations of volatile compounds, such as acids and alcohols. Notably, WHSGF/F and WLSGF/F sensors are sensitive to aldehydes, which are key contributors to lard’s flavor profile [50].

Further, an OPLS-DA analysis was conducted on the aroma sensor response data for lard prepared at different temperatures. As depicted in Figure 5B, the initial two principal components (PC1 at 92.3% and PC2 at 4.8%) accounted for 97.1% of the total variance, indicating that the model efficiently encapsulated the comprehensive information within the sample. The OPLS-DA plot separates DL and WL samples along the PC1 axis, with samples clustering distinctly in different regions, indicating significant taste differences between the two processing methods. These findings confirm that the electronic nose can effectively distinguish lard processed under varying temperature conditions.

### 3.5. Lipid Composition Analysis

**Figure 6:**
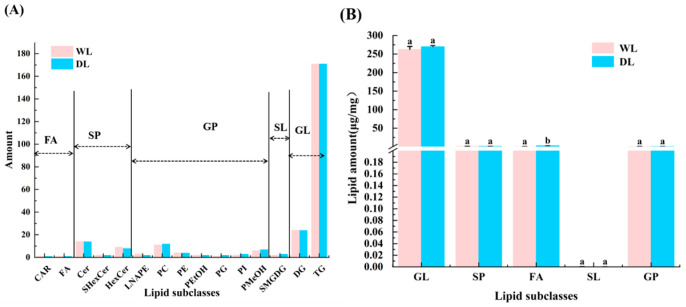
Lipid profiles in WL and DL. (**A**) Proportional composition of lipid classes. (**B**) Lipid subclass. Different letters indicate significant differences (*p* < 0.05).

UPLC-Q-TOF-MS was employed to analyze the lipid profiles of lard prepared at 130 °C (DL) and 100 °C (WL). The results, summarized in Figure 6A, classified the identified lipids into five major categories. The WL sample (100 °C) contained a total of 253 lipid molecules, including 195 glycerolipids (GLs)—with 171 triacylglycerols (TGs) and 24 diacylglycerols (DGs); 29 glycerophospholipids (GPs), comprising 11 phosphatidylcholines (PCs), 4 phosphatidylethanolamines (PEs), 2 phosphatidylinositols (PIs), 1 phosphatidylglycerol (PG), and 3 N-acyl-lysophosphatidylethanolamines (LNAPEs); 2 phosphatidylethanols (PEtOHs); 6 phosphatidylmethanols (PMeOHs); 25 sphingolipids (SPs), including 14 ceramides (Cers), 9 hexosylceramides (HexCers), and 2 sulfated hexosylceramides (SHexCers); 2 glycolipids (SLs); and 2 free fatty acids (FFAs).

In contrast, the DL sample (130 °C) contained 256 lipid molecules: 195 GLs (171 TGs and 24 DGs); 32 GPs (12 PCs, 4 PEs, 3 PIs, 1 PG, and 2 LNAPEs); 2 PEtOHs; 7 PMeOHs; and 24 SPs (14 Cers, 8 HexCers, and 2 SHexCers); 3 SLs; and 2 FFAs. The relative proportions of lipid classes in WL were GLs (77.10%), GPs (11.46%), SPs (9.88%), SLs (0.79%), and FFAs (0.79%), compared with DL proportions of GLs (76.2%), GPs (12.5%), SPs (9.37%), SLs (1.17%), and FFAs (0.78%). TGs and DGs were the predominant lipid molecules in both samples. Quantitative analysis (Figure 6B) revealed significant differences (*p* < 0.05) in the concentration of FFAs between the two samples, while no significant differences were observed in the total amounts of other lipid classes. Among TGs, 21 species exceeded 1% relative abundance, with the six most abundant species being: TG (16:1-16:1-18:0), TG (16:0-16:0-18:1), TG (16:0-16:0-20:4), TG (16:0-18:1-18:2), TG (16:0-16:0-20:1), and TG (18:1-18:2-18:2). The dominant fatty acids in these glycerides were stearic acid, palmitic acid, linoleic acid, and oleic acid, consistent with the known fatty acid composition of lard [33].

**Figure 7:**
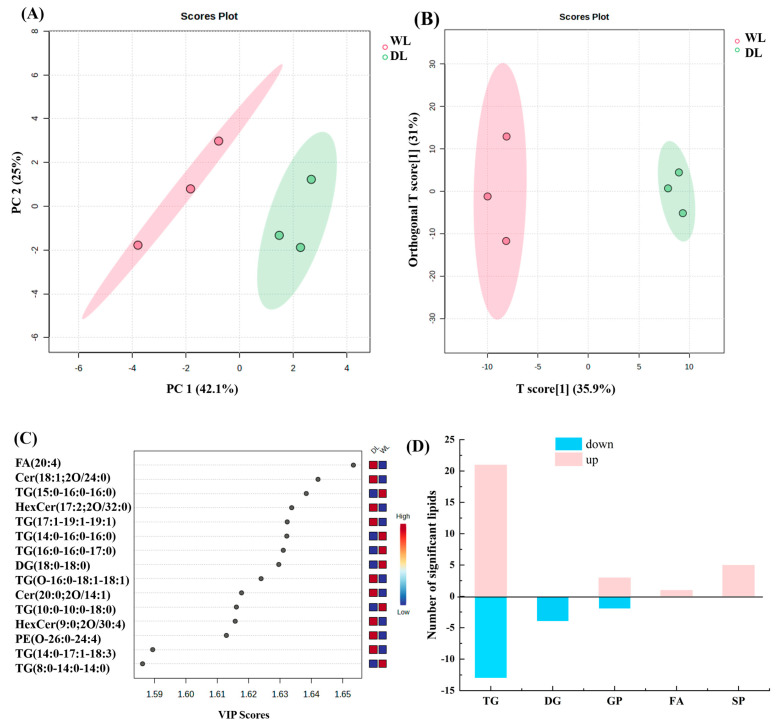
Lipid profiles in WL and DL. (**A**) PCA score chart. (**B**) OPLS-DA score plots. (**C**) Score plot of VIP. (**D**) The number of differentially expressed lipid molecules. “Up” means lipid molecules in DL are more concentrated than in WL whereas “down” means they are less concentrated.

PCA is a statistical method that extracts key features (principal components) from data, effectively reducing dimensionality while capturing the majority of variance in the dataset. It was found that the first two principal components accounted for 42.1% and 25% of the total variance in the initial lipid dataset (Figure 7A). To identify significantly different lipid molecules between WL and DL, both t-tests (*p* < 0.05) and OPLS-DA (VIP > 1) were applied. VIP scores were computed from the OPLS-DA algorithm to prioritize significant lipid molecules, combined with ANOVA results (*p* < 0.05) and VIP > 1 criteria, these 49 lipid biomarkers were identified and chosen for their notable ability to distinguish between groups, demonstrating substantial discriminative power in the analysis.

Among 49 types of lipids, 34 types of TGs, 4 types of DGs, 5 types of GPs, 1 type of FAs, and 5 types of SPs were detected, with Figure 7C only presenting those differential lipids with VIP > 1.586 (including 15 lipids). Compared to WL, lipids marked as “up” indicate higher abundance in DL, while “down” indicates lower abundance in DL relative to WL. The analysis revealed that 30 lipid molecules were significantly elevated in DL, whereas 19 were lower (Figure 7D).

To further characterize the 49 differential lipids, the proportions of each lipid subclass were calculated, focusing on TG, DG, FA, SP, and GP categories. Among the TGs, eight species—TG (16:1-16:1-18:0), TG (18:0-18:1-18:1), TG (17:2-18:1-18:1), TG (18:1-18:1-20:1), TG (16:1-17:1-18:1), TG (16:0-16:0-16:0), TG (17:1-19:1-19:1), and TG (10:0-16:0-18:0)—were the major contributors, accounting for 87.19% and 85.80% of the differences observed in WL and DL, respectively. Within DGs, DG (18:0-18:0) was the predominant species, explaining 61.3% of DG variation in WL and 59.2% in DL.

### 3.6. Correlation Analysis of VOCs and Lipids

**Figure 8:**
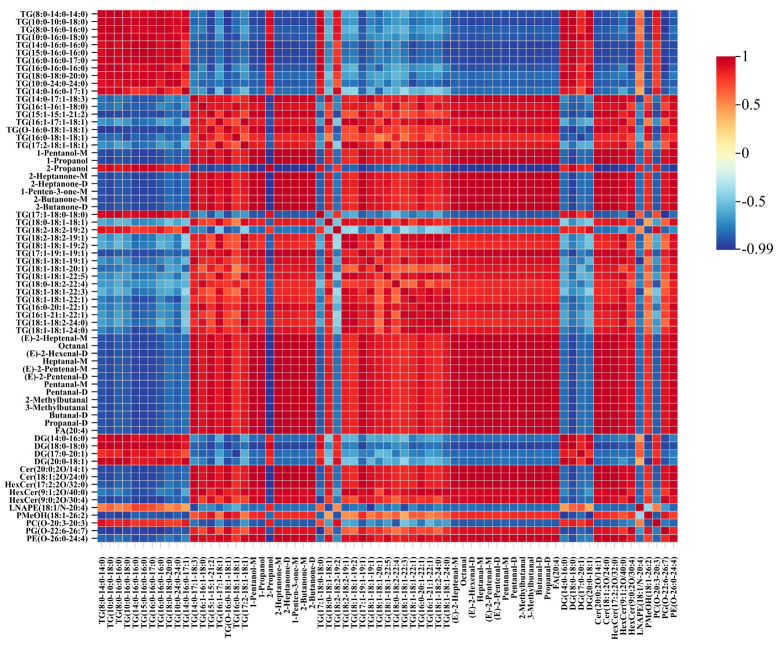
Correlation analysis between discriminatory lipids and signature VOCs.

In this study, a correlation analysis was conducted between 20 differential aroma compounds identified by HS-GC-IMS and 49 lipid molecules screened from lipidomics data. As shown in Figure 8, the TG molecules were categorized into 10 saturated and 24 unsaturated species. These included 2 mono-unsaturated di-saturated TGs (TG (14:0-16:0-17:1), TG (17:1-18:0-18:0)), 10 biunsaturated monosaturated TGs (e.g., TG (14:0-17:1-18:3), TG (16:1-16:1-18:0), TG (O-16:0-18:1-18:1), TG (18:0-18:1-18:1), TG (16:0-18:1-18:1), TG (18:0-18:2-22:4), TG (16:0-20:1-22:1), TG (18:1-18:2-24:0), TG (18:1-18:1-24:0), TG (18:1-18:1-20:1)), and 12 triunsaturated TGs (e.g., TG (18:2-18:2-19:2), TG (18:2-18:2-19:1), TG (18:1-18:1-19:2), TG (17:1-19:1-19:1), TG (18:1-18:1-22:5), TG (16:1-21:1-22:1), and TG (15:1-15:1-21:2)).

For 20 differential aroma compounds, 19 differential aroma compounds (excluding 2-propanol) exhibited a significant negative correlation with saturated TGs. For aldehydes, such as (E)-2-heptenal, octanal, heptanal-M, (E)-2-hexenal-D, (E)-2-pentenal M(D), pentanal-M(D), 3-methylbutanal,2-methylbutanal, butyral-D, and propanal-D showed positive correlations with 21 unsaturated TGs (except TG (14:0-16:0-17:1)). This is consistent with the previous introduction that TG (16:0-18:1-18:1), TG (16:0-18:1-18:2), and TG (16:0-16:1-18:1) are key substrates for the formation of beef flavor [23].

Alcohols (e.g., pentanol and 1-propanol) were also positively associated with unsaturated TGs, excluding TG (18:0-18:1-18:1), TG (17:1-18:0-18:0), and TG (18:2-18:2-19:2). Similarly, ketones were positively correlated with most unsaturated TGs, except TG (17:1-18:0-18:0) and TG (18:2-18:2-19:2). The top four unsaturated TGs showing strong correlations were TG (16:1-16:1-18:0), TG (17:2-18:1-18:1), TG (16:1-17:1-18:1), and TG (18:1-18:1-20:1), all containing key unsaturated fatty acids C16:1 and C18:1. Beyond TGs, ketones and alcohols also showed positive correlations with sphingolipids and phospholipids, including FA (20:4), Cer( 20:0; 2O/14:1), Cer (18:1; 2O/24:0), HexCer (17:2; 2O/32:0), HexCer (9:1; 2O/40:0), HexCer (9:0; 2O/30:4), PMeOH (18:1-26:2), PG (O-22:6-26:7), and PE (O-26:0-24:4). These lipid classes also contain unsaturated fatty acids. These findings demonstrate that the differential volatile aroma compounds are closely associated with triglycerides, glycerophospholipids, and fatty acids, especially those containing unsaturated fatty acid chains. This once again confirms the importance of unsaturated fatty acids in the production of aroma compounds [39].

**Figure 9:**
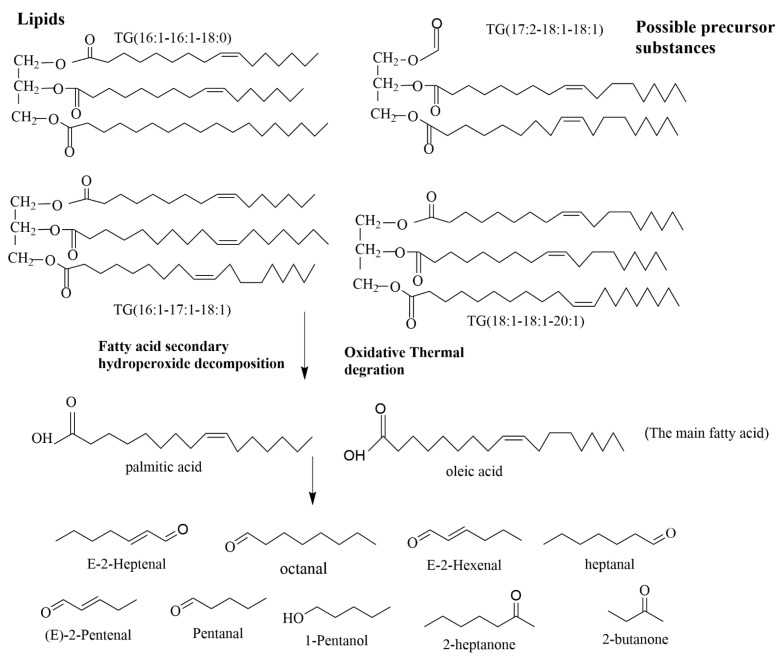
Formation pathway of the typical VOCs from the degradation of the main lipids.

Fat oxidation plays a crucial role in the formation of volatile compounds during oil processing and production. The degradation pathways of key triglycerides—TG (16:1-16:1-18:0), TG (17:2-18:1-18:1), TG (16:1-17:1-18:1), and TG (18:1-18:1-20:1)—are illustrated in Figure 9. These major TG molecules break down their constituent fatty acids C18:1, C16:1, and C17:2, with C16:1 and C18:1 being the primary unsaturated fatty acids in lard. These unsaturated fatty acids undergo oxidation to form hydroperoxides [51]. Hydroperoxides are chemically unstable, and the cleavage of their peroxy bonds (-O–O-) generates reactive alkoxy and hydroxyl radicals. These radicals initiate a cascade of reactions that cleave fatty acid chains, producing various volatile compounds, such as aldehydes—(E)-2-heptenal, octanal, (E)-2-hexenal, heptanal, (E)-2-pentenal, pentanal—alcohols like 1-pentanol, and ketones including 2-heptanone and 2-butanone.

## 4. Conclusions

This study systematically analyzed the differential lipid composition and volatile flavors of lard processed at either 100 °C (WL) or 130 °C (DL). The lipidomic analysis identified TG and DG as the primary lipid components. Using OPLS-DA, 49 significantly different lipids were identified, with TG as the predominant class. Volatile flavor profiling via HS-GC-IMS revealed 67 volatile compounds, mainly aldehydes, alcohols, and acids. OPLS-DA further pinpointed 20 differential volatiles, including 3 alcohols, 12 aldehydes, and 5 ketones. The correlation analysis highlighted that TGs containing unsaturated fatty acids—such as TG (16:1-16:1-18:0), TG (17:2-18:1-18:1), TG (16:1-17:1-18:1), and TG (18:1-18:1-20:1)—serve as key precursors driving flavor differences. This study confirmed the oxidative degradation of C18:1 and C16:1 during high-temperature processing, elucidating the mechanism of lard flavor formation under varying thermal conditions.

## Figures and Tables

**Table 1:** Determination results for AV, PV, and water content of DL and WL.

Classification	WL	DL
PV (g/100 g)	0.066 ± 0.001	0.083 ± 0.003 *
AV (mg KOH/g)	0.675 ± 0.010	0.895 ± 0.033 *
Water content (%)	0.12 ± 0.0009	0.11 ± 0.04

Note: * indicate significant differences at *p* < 0.05. DL: dry rendering lard at 130 °C; WL: wet rendering lard at 100 °C.

**Table 2:** The volatile compound identifications via GC-IMS of WL and DL.

						WL	DL
No	Compound	Formula	MW	RI	Rt (s)	Concentration (μg/100 g Lard)	
1	(E)-2-Nonenal	C_9_H_16_O	140.2	1577	1479.388	13.38 ± 1.89	17.81 ± 1.28 *
2	Benzaldehyde	C_7_H_6_O	106.1	1551.1	1398.721	11.06 ± 0.44	16.11 ± 0.28 ***
3	(E,E)-2,4-Heptadienal	C_7_H_10_O	110.2	1520.2	1308.643	11.07 ± 0.46	13.04 ± 0.27 **
4	(E)-2-Octenal	C_8_H_14_O	126.2	1442.8	1106.975	8.94 ± 0.49	20.31 ± 0.31 ***
5	Nonanal	C_9_H_18_O	142.2	1407.6	1025.979	9.77 ± 0.71	25.4 ± 0.58 ***
6	(E)-2-Heptenal-M	C_7_H_12_O	112.2	1334.6	876.241	34.64 ± 0.75	63.05 ± 0.47 ***
7	(E)-2-Heptenal-D	C_7_H_12_O	112.2	1334.3	875.567	3.83 ± 0.43	9.06 ± 0.44 ***
8	Octanal	C_8_H_16_O	128.2	1302.5	817.541	4.43 ± 0.35	17.39 ± 0.33 ***
9	(E)-2-Hexenal-M	C_6_H_10_O	98.1	1234	710.356	27.87 ± 0.69	40.71 ± 0.45 ***
10	(E)-2-Hexenal-D	C_6_H_10_O	98.1	1234	710.356	2.02 ± 0.05	4.68 ± 0.06 ***
11	Heptanal-M	C_7_H_14_O	114.2	1198.8	661.26	17.6 ± 0.41	52.88 ± 0.41 ***
12	Heptanal-D	C_7_H_14_O	114.2	1199.2	661.8	1.84 ± 0.25	11.96 ± 0.09 ***
13	3-Methyl-2-butenal	C_5_H_8_O	84.1	1192.8	653.168	13.38 ± 0.89 *	11.66 ± 0.51
14	(E)-2-Pentenal-M	C_5_H_8_O	84.1	1150.6	565.767	50.21 ± 0.62	65.14 ± 0.34 ***
15	(E)-2-Pentenal-D	C_5_H_8_O	84.1	1151.4	567.386	7.9 ± 0.28	14.75 ± 0.15 ***
16	Hexanal-M	C_6_H_12_O	100.2	1103.5	481.604	122.05 ± 7.19	189.77 ± 2.18 ***
17	Hexanal-D	C_6_H_12_O	100.2	1103.9	482.143	41.26 ± 8.98	122.53 ± 8.9 ***
18	Pentanal-M	C_5_H_10_O	86.1	1003.6	359.674	76.66 ± 0.72	100.04 ± 1.06 ***
19	Pentanal-D	C_5_H_10_O	86.1	1003.4	359.515	55.24 ± 1.36	141.32 ± 2.23 ***
20	2-Methylbutanal	C_5_H_10_O	86.1	932	306.038	5.59 ± 0.1	18.21 ± 0.65 ***
21	3-Methylbutanal	C_5_H_10_O	86.1	931.4	305.624	0.69 ± 0.04	16.84 ± 0.66 ***
22	Butanal-M	C_4_H_8_O	72.1	890.6	279.539	22.41 ± 0.71	39.11 ± 1.3 ***
23	Butanal-D	C_4_H_8_O	72.1	890.9	279.746	7.74 ± 0.22	20.79 ± 0.66 ***
24	2-Propenal	C_3_H_4_O	56.1	864.4	264.013	89.84 ± 0.48 ***	61.07 ± 1.54
25	Propanal-M	C_3_H_6_O	58.1	818.3	238.756	87.07 ± 1.02	96.3 ± 0.76 ***
26	Propanal-D	C_3_H_6_O	58.1	818.7	238.963	96.24 ± 2.26	165.59 ± 2.68 ***
27	Acetaldehyde	C_2_H_4_O	44.1	764	212.05	102.6 ± 2.26	117.75 ± 3.65 **
	Aldehydes	Subtotal				925.33 ± 9.09	1473.25 ± 11.8
28	Propanoic acid	C_3_H_6_O_2_	74.1	1637.6	1686.433	47.27 ± 6.19	64.19 ± 12.14
29	Acetic acid	C_2_H_4_O_2_	60.1	1505.8	1268.309	265.5 ± 18.64	297.41 ± 19.08
	Acids	Subtotal				312.77 ± 22.07	361.59 ± 11.96
30	1-Octen-3-ol	C_8_H_16_O	128.2	1490.3	1226.631	4.36 ± 0.69	8.58 ± 0.56 **
31	2-Ethyl-1-hexanol	C_8_H_18_O	130.2	1544.8	1379.898	11.72 ± 1.07	12.74 ± 0.74
32	1-Heptanol	C_7_H_16_O	116.2	1490.3	1226.631	8.35 ± 0.69	11.77 ± 0.16 **
33	Tetrahydrolinalool	C_10_H_22_O	158.3	1422.3	1059.045	3.04 ± 0.7	7.18 ± 0.31 ***
34	1-Hexanol	C_6_H_14_O	102.2	1373.7	953.416	1.89 ± 0.15	4.49 ± 0.71 **
35	1-Pentanol-M	C_5_H_12_O	88.1	1268.1	761.54	12.03 ± 0.1	45.22 ± 0.64 ***
36	1-Pentanol-D	C_5_H_12_O	88.1	1267.8	761.07	1.54 ± 0.15	5.65 ± 0.14 ***
37	1-Penten-3-ol	C_5_H_10_O	86.1	1177.4	620.258	35.47 ± 0.86	40.35 ± 0.34 ***
38	1-Butanol-M	C_4_H_10_O	74.1	1162.3	588.966	26.82 ± 0.62	37.64 ± 0.51 ***
39	1-Butanol-D	C_4_H_10_O	74.1	1162.5	589.506	1.39 ± 0.12	2.46 ± 0.28 **
40	1-Propanol	C_3_H_8_O	60.1	1057.4	420.099	7.1 ± 0.15	12.09 ± 0.08 ***
41	Ethanol-M	C_2_H_6_O	46.1	947.4	316.489	181.66 ± 0.48	185.24 ± 1.61 *
42	Ethanol-D	C_2_H_6_O	46.1	946.7	315.983	228.47 ± 3.59	232.53 ± 0.76
43	2-Propanol	C_3_H_8_O	60.1	934.5	307.694	36.14 ± 1.04 ***	19.8 ± 0.43
44	3-Methyl 1-butanol	C_5_H_12_O	88.1	1222.7	694.258	1.54 ± 0.37	1.66 ± 0.04
45	Methanol	CH_4_O	32	908.4	290.658	84.37 ± 3.97	84.17 ± 6.82
	Alcohols	Subtotal				645.86 ± 3.73	711.57 ± 7.95
46	2-Ethylpyrazine	C_6_H_8_N_2_	108.1	1354.1	913.921	2.71 ± 0.12	3.22 ± 0.02 **
47	2-Pentylfuran	C_9_H_14_O	138.2	1245.8	727.62	2.5 ± 0.21	5.06 ± 0.16 ***
	Heterocyclic compounds	Subtotal				5.21 ± 0.23	8.28 ± 0.16
48	1-Hydroxy-2-propanone	C_3_H_6_O_2_	74.1	1317.9	845.205	4.51 ± 0.6	7.74 ± 0.44 **
49	2-Heptanone-M	C_7_H_14_O	114.2	1195.2	656.405	6.67 ± 0.21	20.98 ± 0.17 ***
50	2-Heptanone-D	C_7_H_14_O	114.2	1195.2	656.405	0.82 ± 0.06	2.76 ± 0.14 ***
51	4-Methyl-3-penten-2-one	C_6_H_10_O	98.1	1129.5	526.383	7.61 ± 0.04	8.74 ± 0.19 ***
52	1-Penten-3-one-M	C_5_H_8_O	84.1	1044.7	404.993	9.67 ± 0.09	13.83 ± 0.07 ***
53	1-Penten-3-one-D	C_5_H_8_O	84.1	1044.2	404.454	0.97 ± 0.03	1.67 ± 0.1 ***
54	2-Pentanone	C_5_H_10_O	86.1	999.5	355.466	5.45 ± 0.05	6.82 ± 0.16 ***
55	2-Butanone-M	C_4_H_8_O	72.1	915.3	295.066	24.27 ± 0.27	31.45 ± 0.22 ***
56	2-Butanone-D	C_4_H_8_O	72.1	915.6	295.273	3.91 ± 0.1	8.11 ± 0.2 ***
57	Acetone	C_3_H_6_O	58.1	837	248.693	29.66 ± 0.89	42.92 ± 0.88 ***
58	3-Hydroxy-2-butanone	C_4_H_8_O_2_	88.1	1301.9	816.467	2.47 ± 0.38	3.6 ± 0.26 *
59	2,3-Butanedione	C_4_H_6_O_2_	86.1	994.1	350.47	1.38 ± 0.05	1.95 ± 0.19 **
	Ketones	Subtotal				97.37 ± 0.67	150.56 ± 1.41
60	Ethyl butanoate	C_6_H_12_O_2_	116.2	1040.5	400.138	5.87 ± 0.18 *	5.27 ± 0.22
61	Ethyl acetate-M	C_4_H_8_O_2_	88.1	897.6	283.887	29.55 ± 1.55 **	24.41 ± 0.66
62	Ethyl acetate-D	C_4_H_8_O_2_	88.1	897.6	283.887	10.16 ± 0.86 **	7.45 ± 0.26
	Esters	Subtotal				45.58 ± 2.55	37.12 ± 0.73
63	Thiophene	C_4_H_4_S	84.1	1033	391.505	137.69 ± 1.47	136.68 ± 0.44
64	Toluene	C_7_H_8_	92.1	1066.7	431.491	24.36 ± 0.25	27.96 ± 0.43 ***
65	1	*	0	1067.1	431.969	5.73 ± 0.1	6.7 ± 0.06 ***
66	2	*	0	1206.6	671.868	0.82 ± 0.11	1.45 ± 0.08 **
67	3	*	0	1170.4	605.673	1.95 ± 0.07	2.89 ± 0.08 ***
	Others	Subtotal				170.55 ± 1.56	175.69 ± 0.92
		Total				2202.67 ± 26.6	2918.06 ± 22.35

The values are the means ± standard deviation; *: *p* < 0.05; **: *p* < 0.01; and ***: *p* < 0.001.

## Data Availability

The original contributions presented in this study are included in the article/Supplementary Materials. Further inquiries can be directed to the corresponding authors.

## Supplementary Materials

The following supporting information can be downloaded at: https://www.mdpi.com/article/10.3390/foods14142441/s1, Table S1. Sensitive substances corresponding to various electronic nose sensors. Table S2. Composition of the main TG and DG lipids in lard.
