# Supplementary material for: Formation Mechanism of Lipid and Flavor of Lard Under the Intervention of Heating Temperature via UPLC-TOF-MS/MS with OPLS-DA and HS-GC-IMS Analysis
Source: Foods. 2025 Jul 11;14(14):2441. doi: 10.3390/foods14142441 (PMC12295278; doi:10.3390/foods14142441)
Supplement: Supplementary file 1 [file foods-14-02441-s001.zip › foods-3740527-supplementary.pdf]

Table S1. Sensitive substances corresponding to various electronic nose sensors

Sensor	Performance Description
WLSGF/A	Solvents
WLSGF/B	Hydrocarbons，Propane/butane，Methane，Hydrogen
WLSGF/C	Methane
WLSGF/D	Fluorur
WLSGF/E	Aromatic compounds，General purpose，Humidity，Combustion gas monitoring
WLSGF/F	Hydrogen，Aldehydes，Alcohol，Aromatic compounds，Ammonia,Amines，Nitrogen oxide，General air pollution，Monitoring，Cigarette smoke
WMSGF/A	Hydrocarbons，Solvents，Combustion gas Monitoring，
WMSGF/B	/
WMSGF/C	Alcohol，Combustion gas monitoring
WMSGF/D	Ammonia,Amines，Fluorur
WMSGF/E	Hydrogen sulfide，Nitrogen oxide，Combustion gas monitoring
WMSGF/F	Alcohol，General air pollution，Monitoring，Carbon monoxide and gas monitoring
WHSGF/A	Fluorur，Nitrogen oxide，Ozone
WHSGF/B	Ammonia,Amines，Carbon monoxide，Carbon monoxide and gas monitoring
WHSGF/C	Alcohol
WHSGF/D	Ammonia,Amines
WHSGF/E	Hydrogen sulfide
WHSGF/F	Propane/butane，Aldehydes

Table S2 Composition of the main TG and DG lipids in lard

No	m/z	RT	Lipid Name	Adduct Type	WL（μg/mg）	DL（μg/mg）
1	1014.942	638.5	TG 18:1-18:1-26:1	[M+NH4]+	0.011±0.001	0.011±0
2	1016.954	647.5	TG 18:1-18:1-26:0	[M+NH4]+	0.012±0.007	0.008±0
3	1018.973	656.9	TG 16:0-18:1-28:0	[M+NH4]+	0.009±0.005	0.006±0
4	656.5813	452.7	TG 8:0-14:0-14:0	[M+NH4]+	0.011±0.002*	0.005±0
5	682.5985	455.1	TG 8:0-14:0-16:1	[M+NH4]+	0.023±0.002	0.023±0.001
6	684.6108	470.5	TG 10:0-10:0-18:0	[M+NH4]+	0.032±0.002*	0.025±0.001
7	708.612	456.8	TG 8:0-16:1-16:1	[M+NH4]+	0.039±0.003	0.042±0.002
8	710.6263	471.3	TG 10:0-14:0-16:1	[M+NH4]+	0.076±0.006	0.073±0.003
9	712.6428	486	TG 8:0-16:0-16:0	[M+NH4]+	0.1±0.008*	0.083±0.002
10	724.6463	479.1	TG 13:1-14:0-14:0	[M+NH4]+	0.014±0.001	0.014±0.001
11	734.6264	459.1	TG 8:0-8:0-26:3	[M+NH4]+	0.036±0.003	0.038±0.002
12	736.6443	473.1	TG 14:0-14:1-14:1	[M+NH4]+	0.197±0.016	0.217±0.011
13	738.6584	486.7	TG 10:0-16:0-16:1	[M+NH4]+	0.286±0.026	0.286±0.008
14	740.6751	500.8	TG 14:0-14:0-14:0	[M+NH4]+	0.296±0.018	0.259±0.003
15	750.6612	480.5	TG 9:0-17:1-17:1	[M+NH4]+	0.018±0.001	0.02±0.001
16	752.6774	494.3	TG 9:0-17:0-17:1	[M+NH4]+	0.015±0.001	0.015±0
17	762.6615	474.6	TG 8:0-18:1-18:2	[M+NH4]+	0.235±0.016	0.257±0.008
18	764.6758	488.1	TG 8:0-18:1-18:1	[M+NH4]+	1.008±0.085	1.047±0.019
19	766.6918	501.8	TG 10:0-16:0-18:1	[M+NH4]+	1.132±0.06	1.093±0.022
20	768.7057	517.2	TG 10:0-16:0-18:0	[M+NH4]+	0.582±0.036*	0.482±0.019
21	776.6776	482	TG 9:0-18:1-18:2	[M+NH4]+	0.017±0.001	0.017±0.001
22	778.6892	495.3	TG 9:0-18:1-18:1	[M+NH4]+	0.052±0.004	0.054±0.002
23	780.7074	510.1	TG 15:0-15:0-15:1	[M+NH4]+	0.033±0.003	0.031±0
24	786.6538	458.3	TG 10:0-10:0-26:5	[M+NH4]+	0.001±0	0.001±0
25	788.6754	476.4	TG 10:0-18:2-18:2	[M+NH4]+	0.302±0.024	0.308±0.006
26	790.6852	475.6	TG 10:0-18:1-18:2	[M+NH4]+	0.08±0.004	0.084±0.002
27	790.6895	489.5	TG 10:0-10:0-26:3	[M+NH4]+	1.185±0.073	1.228±0.038

28	792.699	489.1	TG 14:0-14:0-18:2	[M+NH4]+	0.268±0.013	0.274±0.012
29	794.7224	517.9	TG 12:0-16:1-18:0	[M+NH4]+	2.19±0.128	2.057±0.085
30	794.7233	530.6	TG 10:0-10:0-26:1	[M+NH4]+	0.031±0.003	0.029±0.002
31	796.7374	534.7	TG 14:0-16:0-16:0	[M+NH4]+	0.448±0.018*	0.372±0.01
32	804.7075	497	TG 15:1-16:1-16:1	[M+NH4]+	0.028±0.002	0.031±0.001
33	806.7195	473.1	TG 15:1-15:1-17:0	[M+NH4]+	0.021±0.001	0.023±0.002
34	806.7215	511.2	TG 15:0-16:1-16:1	[M+NH4]+	0.072±0.003	0.075±0.001
35	808.7379	486.6	TG 13:0-13:0-21:1	[M+NH4]+	0.058±0.005	0.061±0.002
36	808.7372	527.1	TG 14:0-16:0-17:1	[M+NH4]+	0.111±0.006*	0.101±0.001
37	810.7505	501.1	TG 13:0-13:0-21:0	[M+NH4]+	0.036±0.002	0.032±0
38	810.7508	544.9	TG 15:0-16:0-16:0	[M+NH4]+	0.046±0.003*	0.033±0.002
39	814.6929	479.4	TG 10:0-10:0-28:5	[M+NH4]+	0.038±0.002	0.041±0.002
40	816.7067	491.4	TG 12:0-18:2-18:2	[M+NH4]+	0.398±0.017	0.416±0.013
41	818.7211	504.6	TG 16:1-16:1-16:1	[M+NH4]+	1.965±0.137	2.03±0.026
42	820.7309	504	TG 14:0-16:0-18:2	[M+NH4]+	0.605±0.043	0.624±0.011
43	820.7361	519	TG 14:1-16:0-18:1	[M+NH4]+	6.293±0.242	6.049±0.113
44	822.7512	486.6	TG 16:0-16:0-16:1	[M+NH4]+	0.053±0.004	0.054±0.001
45	822.7438	519	TG 14:0-16:1-18:0	[M+NH4]+	1.337±0.063	1.329±0.026
46	824.7674	554.6	TG 16:0-16:0-16:0	[M+NH4]+	0.973±0.072*	0.766±0.035
47	830.7208	499.4	TG 14:0-17:1-18:3	[M+NH4]+	0.029±0.001	0.033±0*
48	832.7371	474.6	TG 15:1-15:1-19:1	[M+NH4]+	0.023±0.001	0.026±0.003
49	832.7366	512.9	TG 14:0-17:1-18:2	[M+NH4]+	0.187±0.012	0.202±0.004
50	834.7526	528.2	TG 15:0-17:1-17:1	[M+NH4]+	0.562±0.034	0.597±0.015
51	834.7901	571.3	TG O-18:2-16:0-16:0	[M+NH4]+	0.021±0.002	0.025±0.001
52	836.769	501.9	TG 15:0-15:0-19:1	[M+NH4]+	0.227±0.014	0.216±0.009
53	836.7688	545.8	TG 15:0-17:0-17:1	[M+NH4]+	0.819±0.048	0.778±0.011
54	836.8064	594.6	TG O-18:1-16:0-16:0	[M+NH4]+	0.043±0.002	0.045±0.002
55	838.785	566.7	TG 16:0-16:0-17:0	[M+NH4]+	0.222±0.011*	0.176±0.006
56	838.8231	619.9	TG O-18:0-16:0-16:0	[M+NH4]+	0.009±0.001	0.009±0
57	840.7054	487.1	TG 10:0-18:1-22:5	[M+NH4]+	0.015±0.003	0.017±0.004
58	842.721	494.3	TG 14:1-18:2-18:2	[M+NH4]+	0.366±0.028	0.387±0.01
59	844.7307	493.6	TG 14:1-18:1-18:2	[M+NH4]+	0.149±0.009	0.154±0.005
60	844.7366	506.6	TG 14:0-18:2-18:2	[M+NH4]+	4.019±0.296	4.212±0.128
61	846.7524	520.7	TG 16:0-16:0-18:3	[M+NH4]+	12.097±0.44	12.759±0.185
62	848.7674	488	TG 16:0-16:1-18:1	[M+NH4]+	0.092±0.004	0.099±0.001
63	848.7599	520.5	TG 16:0-16:0-18:2	[M+NH4]+	2.643±0.314	2.584±0.048
64	848.768	536.5	TG 16:1-16:1-18:0	[M+NH4]+	19.861±0.161	20.435±0.174*
65	848.7678	550.8	TG 14:0-18:1-18:1	[M+NH4]+	1.594±0.113	1.508±0.061
66	850.7822	501.8	TG 16:0-16:1-18:0	[M+NH4]+	0.248±0.061	0.199±0.005
67	850.7833	555.6	TG 16:0-16:0-18:1	[M+NH4]+	16.109±0.682	15.374±0.47
68	850.7829	572	TG 14:1-18:0-18:0	[M+NH4]+	4.288±0.198	3.106±1.316
69	852.798	578.2	TG 16:0-16:0-18:0	[M+NH4]+	3.429±0.411	3.055±0.065
70	856.7347	502.5	TG 15:1-18:2-18:2	[M+NH4]+	0.063±0.004	0.068±0.003
71	858.7614	482.4	TG 15:1-15:1-21:2	[M+NH4]+	0.007±0.002	0.02±0.002*
72	858.7479	503.3	TG 15:0-18:2-18:2	[M+NH4]+	0.006±0.002	0.007±0.003
73	858.7552	514.7	TG 16:1-17:2-18:1	[M+NH4]+	0.511±0.045	0.573±0.023
74	860.7608	514.7	TG 15:0-17:1-19:2	[M+NH4]+	0.064±0.048	0.101±0.004
75	860.7683	529.8	TG 16:1-17:1-18:1	[M+NH4]+	1.761±0.072	1.951±0.053*
76	860.8073	571.7	TG O-20:3-16:0-16:0	[M+NH4]+	0.023±0.006	0.031±0.005
77	862.7832	503.2	TG 15:0-15:0-21:2	[M+NH4]+	0.288±0.026	0.293±0.008
78	862.7821	547.1	TG 17:0-17:1-17:1	[M+NH4]+	3.483±0.2	3.656±0.088

79	862.8205	595.3	TG O-16:0-18:1-18:1	[M+NH4]+	0.054±0.001	0.061±0*
80	864.8003	567.7	TG 17:0-17:0-17:1	[M+NH4]+	3.674±0.184	3.632±0.189
81	864.8396	620.1	TG O-16:1-18:0-18:0	[M+NH4]+	0.066±0.003	0.066±0.002
82	866.8048	517.9	TG 17:0-17:0-17:0	[M+NH4]+	0.027±0.002	0.025±0.006
83	868.7369	499.4	TG 16:2-18:1-18:3	[M+NH4]+	0.437±0.024	0.278±0.198
84	870.7536	508.7	TG 16:1-18:2-18:2	[M+NH4]+	6.056±0.349	6.492±0.198
85	870.757	528	TG 16:0-16:1-20:4	[M+NH4]+	0.007±0.001	0.007±0.001
86	872.762	507.7	TG 16:0-18:2-18:2	[M+NH4]+	1.673±0.084	2.05±0.28
87	872.7706	523	TG 16:0-16:0-20:4	[M+NH4]+	27.413±1.106	28.669±0.716
88	874.7868	539.2	TG 16:0-18:1-18:2	[M+NH4]+	38.688±0.362	43.541±4.36
89	876.7929	539.1	TG 16:0-18:0-18:2	[M+NH4]+	9.102±0.249	9.299±0.087
90	876.8014	621.8	TG 16:0-18:1-18:1	[M+NH4]+	0.438±0.186	0.948±0.225*
91	878.8145	517.9	TG 16:0-18:0-18:1	[M+NH4]+	0.295±0.009	0.295±0.017
92	878.8077	557.6	TG 16:0-16:0-20:1	[M+NH4]+	15.411±0.444	14.675±0.951
93	880.8209	580	TG 16:0-18:0-18:0	[M+NH4]+	6.561±0.163	6.469±0.087
94	882.7537	502.9	TG 17:2-18:2-18:2	[M+NH4]+	0.064±0.005	0.069±0.002
95	884.7646	503.1	TG 17:1-18:2-18:2	[M+NH4]+	0.011±0.001	0.011±0.001
96	886.7841	506.4	TG 17:0-18:2-18:2	[M+NH4]+	0.051±0.002	0.053±0.002
97	886.782	530.9	TG 17:2-18:1-18:1	[M+NH4]+	2.018±0.07	2.228±0.08*
98	888.799	548.3	TG 17:1-18:1-18:1	[M+NH4]+	3.542±0.216	3.857±0.166
99	890.8089	531.1	TG 17:0-18:1-18:1	[M+NH4]+	0.01±0.006	0.006±0
100	890.8147	568.7	TG 17:1-17:1-19:0	[M+NH4]+	3.768±0.142	4.028±0.164
101	890.8589	620.7	TG O-22:2-16:0-16:0	[M+NH4]+	0.008±0.002	0.008±0.001
102	892.8247	519	TG 15:1-19:0-19:0	[M+NH4]+	0.083±0.004	0.078±0.008
103	892.8295	534.9	TG 17:1-18:0-18:0	[M+NH4]+	0.473±0.005*	0.457±0.005
104	892.8293	592.9	TG 17:0-17:0-19:1	[M+NH4]+	2.265±0.126	2.282±0.026
105	894.7536	497.5	TG 18:2-18:2-18:3	[M+NH4]+	0.929±0.074	1.046±0.071
106	894.8445	554.1	TG 15:0-19:0-19:0	[M+NH4]+	0.066±0.01	0.056±0.009
107	894.8422	592.7	TG 17:0-17:0-19:0	[M+NH4]+	0.274±0.01	0.283±0.013
108	894.8503	618	TG 17:0-18:0-18:0	[M+NH4]+	0.292±0.021	0.264±0.024
109	896.7651	518	TG 18:2-18:2-18:2	[M+NH4]+	1.463±0.1	1.505±0.091
110	898.7841	524.4	TG 18:1-18:2-18:2	[M+NH4]+	16.708±0.652	17.654±0.581
111	900.795	506.8	TG 18:1-18:1-18:2	[M+NH4]+	0.038±0.003	0.03±0.005
112	902.8179	504.6	TG 18:1-18:1-18:1	[M+NH4]+	0.139±0.012	0.148±0.004
113	904.8313	518.8	TG 18:0-18:0-18:2	[M+NH4]+	0.4±0.013	0.407±0.019
114	904.8238	560.6	TG 18:0-18:1-18:1	[M+NH4]+	3.696±0.113	3.946±0.062*
115	906.8361	519	TG 18:0-18:0-18:1	[M+NH4]+	0.091±0.005	0.094±0.004
116	908.8563	606.5	TG 18:0-18:0-18:0	[M+NH4]+	2.18±0.083	2.139±0.022
117	910.781	525.9	TG 18:2-18:2-19:2	[M+NH4]+	0.046±0.006*	0.032±0.006
118	912.8017	509	TG 17:1-19:2-19:2	[M+NH4]+	0.076±0.001	0.097±0.035
119	912.7995	532.4	TG 18:2-18:2-19:1	[M+NH4]+	0.159±0.009	0.177±0.004*
120	914.8144	506.8	TG 17:0-19:2-19:2	[M+NH4]+	0.326±0.027	0.341±0.011
121	914.8147	549.3	TG 18:1-18:1-19:2	[M+NH4]+	0.436±0.027	0.486±0.015*
122	916.8279	520.5	TG 17:1-19:1-19:1	[M+NH4]+	0.702±0.001	0.761±0.008*
123	916.8284	548.7	TG 18:1-18:1-19:1	[M+NH4]+	0.269±0.012	0.303±0.005*
124	918.8404	520.5	TG 13:0-21:1-21:1	[M+NH4]+	0.143±0.006	0.142±0.002
125	918.8444	568.8	TG 18:0-18:0-19:2	[M+NH4]+	0.454±0.069	0.49±0.062
126	918.8481	593.2	TG 17:0-19:1-19:1	[M+NH4]+	0.401±0.029	0.443±0.006
127	920.7646	505.3	TG 18:2-18:2-20:4	[M+NH4]+	0.09±0.021	0.116±0.007
128	920.8579	536.2	TG 13:1-21:0-21:0	[M+NH4]+	0.227±0.041	0.26±0.007
129	920.8584	592.7	TG 18:0-18:1-19:0	[M+NH4]+	0.718±0.051	0.695±0.018

130	920.8659	618.1	TG 16:0-19:1-20:0	[M+NH4]+	0.302±0.042	0.286±0.019
131	922.7812	41.2	TG 18:1-18:1-20:5	[M+NH4]+	0±0	0±0
132	922.8661	555.1	TG 18:0-18:0-19:0	[M+NH4]+	0.096±0.026	0.076±0.011
133	924.7954	510.3	TG 18:2-18:2-20:2	[M+NH4]+	0.545±0.108	0.593±0.145
134	926.8183	507.5	TG 18:1-18:1-20:3	[M+NH4]+	0.018±0.003	0.023±0.003
135	926.8157	547.6	TG 18:1-18:2-20:2	[M+NH4]+	3.258±1.051	3.182±1.241
136	926.8174	568.5	TG 18:0-18:1-20:4	[M+NH4]+	0.009±0.001	0.004±0.004
137	928.8272	526.3	TG 18:1-18:1-20:2	[M+NH4]+	0.327±0.011	0.263±0.164
138	930.844	583	TG 18:1-18:1-20:1	[M+NH4]+	1.325±0.091	1.548±0.048*
139	932.8592	561.4	TG 18:1-18:1-20:0	[M+NH4]+	0.325±0.086	0.282±0.003
140	934.8785	584.1	TG 18:0-18:0-20:1	[M+NH4]+	0.323±0.029	0.333±0.034
141	936.891	638.3	TG 18:0-18:0-20:0	[M+NH4]+	0.2±0.023*	0.159±0.005
142	942.8441	543.2	TG 18:2-19:1-20:1	[M+NH4]+	0.075±0.009	0.089±0.008
143	944.8629	563.2	TG 19:1-19:1-19:1	[M+NH4]+	0.045±0.015	0.04±0.009
144	946.7808	512.4	TG 18:2-18:2-22:5	[M+NH4]+	0.078±0.009	0.074±0.004
145	946.8756	592.9	TG 18:0-19:1-20:1	[M+NH4]+	0.085±0.031	0.082±0.008
146	946.8777	618.3	TG 17:0-20:1-20:1	[M+NH4]+	0.067±0.007	0.074±0.005
147	948.8029	517.9	TG 18:2-18:2-22:4	[M+NH4]+	0.165±0.011	0.183±0.012
148	948.8932	608.9	TG 19:0-19:0-19:1	[M+NH4]+	0.201±0.034	0.172±0.012
149	948.8924	633.3	TG 16:0-18:1-23:0	[M+NH4]+	0.181±0.062	0.147±0.009
150	950.8159	532.4	TG 18:1-18:1-22:5	[M+NH4]+	0.444±0.022	0.517±0.028*
151	950.9129	626.8	TG 16:0-18:0-23:0	[M+NH4]+	0.26±0.183	0.126±0.011
152	952.8255	532.4	TG 18:0-18:2-22:4	[M+NH4]+	0.187±0.006	0.207±0.01*
153	952.8342	549.1	TG 18:1-18:1-22:4	[M+NH4]+	0.642±0.033	0.698±0.004
154	954.8416	548.5	TG 18:1-18:1-22:3	[M+NH4]+	0.267±0.015	0.298±0.011*
155	954.8505	569.8	TG 18:0-18:1-22:4	[M+NH4]+	0.384±0.052	0.444±0.056
156	956.8637	584	TG 18:0-18:0-22:4	[M+NH4]+	0.082±0.008	0.091±0.007
157	958.8766	606.4	TG 18:1-18:1-22:1	[M+NH4]+	0.152±0.018	0.189±0.01*
158	960.8928	626.4	TG 16:0-20:1-22:1	[M+NH4]+	0.239±0.01	0.266±0.004*
159	962.9079	608.3	TG 18:0-18:0-22:1	[M+NH4]+	0.207±0.026	0.332±0.076
160	962.9075	626.4	TG 16:0-18:1-24:0	[M+NH4]+	0.249±0.017	0.254±0.006
161	964.9226	638.2	TG 18:0-18:0-22:0	[M+NH4]+	0.267±0.078	0.125±0.006
162	964.9285	647.6	TG 10:0-24:0-24:0	[M+NH4]+	0.16±0.033*	0.089±0.008
163	972.8956	619.5	TG 16:1-21:1-22:1	[M+NH4]+	0.014±0.002	0.019±0.002*
164	974.9109	633.2	TG 18:1-18:1-23:0	[M+NH4]+	0.027±0.001	0.029±0.001
165	978.9436	651.6	TG 16:0-18:0-25:0	[M+NH4]+	0.035±0.035	0.01±0.001
166	980.8671	570.2	TG 18:1-18:1-24:4	[M+NH4]+	0.025±0.006	0.028±0.005
167	982.8819	592.3	TG 18:0-18:1-24:4	[M+NH4]+	0.008±0.002	0.012±0.003
168	984.8913	611.5	TG 18:1-18:1-24:2	[M+NH4]+	0.02±0.002	0.023±0.003
169	986.9072	627.2	TG 18:1-18:2-24:0	[M+NH4]+	0.054±0.003	0.059±0.001*
170	988.9202	626.7	TG 18:1-18:1-24:0	[M+NH4]+	0.029±0.002	0.035±0.001*
171	992.9558	648	TG 18:0-18:0-24:0	[M+NH4]+	0.073±0.053	0.021±0.001
172	556.4927	369.5	DG 12:0-18:1	[M+NH4]+	0.015±0.001	0.015±0
173	558.5065	394.4	DG 14:0-16:0	[M+NH4]+	0.017±0.002*	0.013±0.001
174	582.5098	375.2	DG 14:0-18:2	[M+NH4]+	0.108±0.009	0.108±0.004
175	584.5232	396.9	DG 14:0-18:1	[M+NH4]+	0.336±0.021	0.317±0.006
176	606.5074	358.8	DG 16:1-18:3	[M+NH4]+	0.003±0	0.003±0
177	608.5253	379.3	DG 16:1-18:2	[M+NH4]+	0.065±0.006	0.066±0.002
178	610.5394	401.8	DG 16:0-18:2	[M+NH4]+	1.581±0.142	1.484±0.024
179	624.5555	412.4	DG 17:1-18:1	[M+NH4]+	0.037±0.004	0.038±0.002
180	626.5703	431.6	DG 17:0-18:1	[M+NH4]+	0.029±0.003	0.026±0.001

181	632.525	364.5	DG 18:2-18:3	[M+NH4]+	0.015±0.001	0.015±0.001
182	634.5397	384.6	DG 18:2-18:2	[M+NH4]+	0.2±0.019	0.198±0.006
183	634.5394	397	DG 16:0-20:4	[M+NH4]+	0.029±0.002	0.026±0.001
184	636.5467	384.6	DG 18:1-18:2	[M+NH4]+	0.016±0.001	0.022±0.01
185	638.5676	401.8	DG 18:1-18:1	[M+NH4]+	0.322±0.036	0.295±0.022
186	640.5856	441.7	DG 18:0-18:1	[M+NH4]+	1.406±0.247	1.097±0.044
187	642.6037	459.1	DG 18:0-18:0	[M+NH4]+	0.143±0.01*	0.107±0.001
188	654.6026	420.5	DG 17:0-20:1	[M+NH4]+	0.034±0.001*	0.031±0.001
189	658.5403	378.4	DG 18:2-20:4	[M+NH4]+	0.007±0.001	0.007±0
190	660.5531	398.7	DG 18:1-20:4	[M+NH4]+	0.025±0.002	0.025±0.001
191	662.5705	420.1	DG 18:0-20:4	[M+NH4]+	0.075±0.014	0.084±0.023
192	664.5869	425.8	DG 18:1-20:2	[M+NH4]+	0.045±0.004	0.046±0.001
193	666.602	442.7	DG 18:1-20:1	[M+NH4]+	0.058±0.005	0.055±0.003
194	668.6167	460	DG 20:0-18:1	[M+NH4]+	0.04±0.003*	0.034±0.001
195	690.6017	436.2	DG 18:0-22:4	[M+NH4]+	0.012±0.001	0.012±0.001
